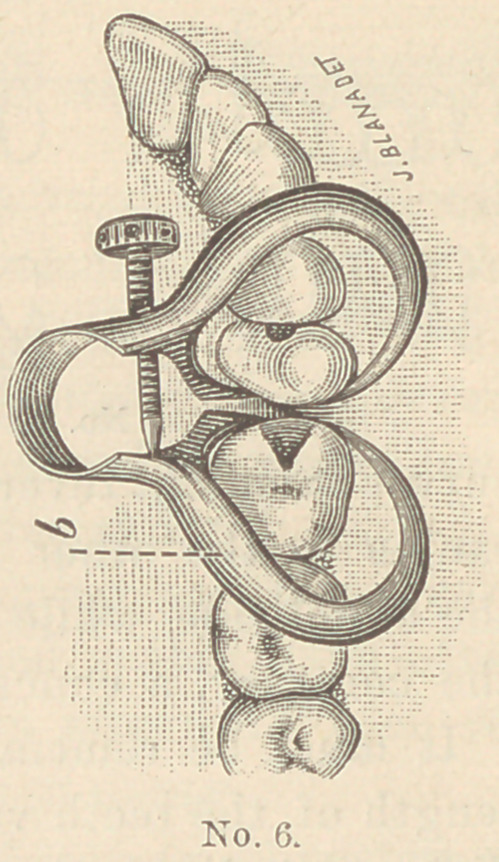# The Facilitation of Contour Work

**Published:** 1885-03

**Authors:** E. A. Bogue


### THE FACILITATION OF CONTOUR WORK.

BY E. A. BOGUE, M. D., D. D. S.

READ BEFORE THE ODONTOLOGICAL SOCIETY OF GREAT BRITAIN.

I believe it is generally conceded that the normal form and position of the human teeth are such as are best calculated to resist the destructive tendencies which surround them.

When, however, decay has attacked the teeth upon their approximal sides, the difficulties of restoration to the normal form have been so great as to discourage many from ever undertaking it.

The consequence has been that for obtaining access to very small cavities of decay, great slots or V-shaped spaces have often been filed between two teeth, which slots have afterward been receptacles for the debris of food and the nidus of decay.

A more careful class of operators have used wedges of cotton or tapes or wood until space was procured, and then placed a wooden wedge at the margin of the gum between the two teeth to be operated upon.

This process is both long and painful, and is not always certain.

Some ten or twelve years ago Dr. Jarvis, of New York, devised an instrument to separate two adjoining teeth by means of a screw, so that an examination could be made or a wedge inserted.

Shortly afterward, Dr. Perry made an improvement upon the form of the instrument, but it was still not applicable to the majority of cases.

With the consent of both these gentlemen I have undertaken to improve the device still further, and this evening take pleasure in presenting for your examination an instrument that I have used for the last four years, with great advantage to myself and an enormous saving of pain and tooth substance to my patients.

I have recently made some further modifications in the form and size of the instrument,that are shown in the accompanying engravings.

It is a question of applying the force gradually, to separate the teeth without touching the gums, in order to obtain little by little the necessary space, and once obtained, to have room enough to work without being hindered by the instrument itself.

If we examine the dental arches of a well developed subject, looking at the maxilla horizontally, we find that the arch of the lower jaw presents three different curves.

The first is represented by a line commencing at the upper end of each canine and describing a curve that is convex towards the upper jaw; the two other curves are indicated by the molars, which present a curve with the concavity looking downwards.

No. 1.

No. 2.

In the upper jaw the same curves exist, but reversed; and we have besides a curve from one canine to the other which is convex on the labial side, while the molars have, on the contrary, a concave curve.

It must be remembered also that the length of the teeth varies considerably in different individuals.

For these various reasons it is easy to understand the difficulties in the way of a perfect adaptation of the instrument to all mouths; hence the advisability of having several to suit different cases.

I enclose several illustrations of the instrument in position upon various teeth.

The one upon the incisors has two small screws to adapt it to the varying lengths of teeth, and so to raise the instrument by letting these small screws press upon the ends of the teeth that the points of the separating claws shall not impinge upon the gums.

No. 3.

The one showing the cavities in the molar and bicuspid is shaped so as to rest upon the ends of the teeth, and the pointed ends of the claw are just the length of a short tooth below the middle of the bow.

The last illustration shows a wider bow further removed from the cavity to be operated upon, out of the way of all instruments and with the points of the claws a little further from the middle

No. 4.

of the bow, thus adapting it to lower back teeth and to long upper teeth.

The distance between the points of the claws varies according to the class of teeth it is to be used upon.

For incisors the points would have to be from 4 to 5 millimeters apart, while for molars they might need to be 7 or 9 millimeters, or even more in some cases.

No. 5.

No. 6.

I find that in many cases, if the teeth to be operated upon can be separated with cotton or tape for one or two or more days before the operation, the screw separator being then applied holds them steadily and firmly while the operation is being performed, and so painlessly that patients often go to sleep during the operation.

Of course one is enabled to go on much more rapidly and with less fatigue to both parties.

When the fillings are in, another turn is given to the screw, to obtain room to finish the cervical margins by means of Dr. Smith's disks, that cut only upon the outer margin, and by the use of polishing tapes and sharp lancets.

Upon taking off the separator there remain two knuckles of gold or other filling that touch each other, like two apples side by side, over which the floss silk and the brush can pass readily to completely cleanse the entire circumference of the filling, while these two contiguous fillings, restoring the original or the ideal form of the tooth, effectually prevent the disagreeable or painful crowding of food between the teeth and under the gums to their detriment.

Since writing this article, and after it was in type, Dr. Perry has for the first time showed me his improved separator. I find that intended for the incisor teeth such a marked improvement upon my own form, that I desire most emphatically to acknowledge its superiority.

## Figures and Tables

**No. 1. f1:**
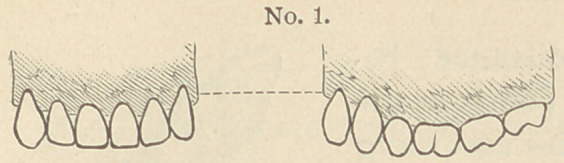


**No. 2. f2:**
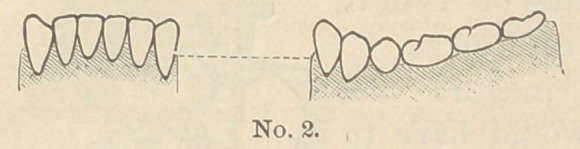


**No. 3. f3:**
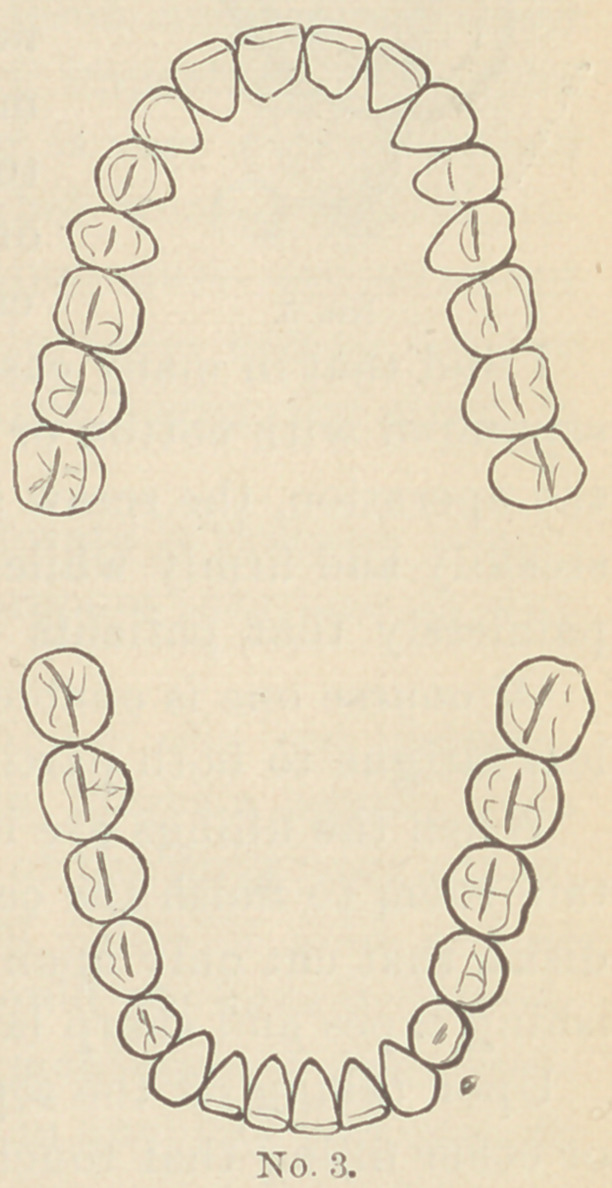


**No. 4. f4:**
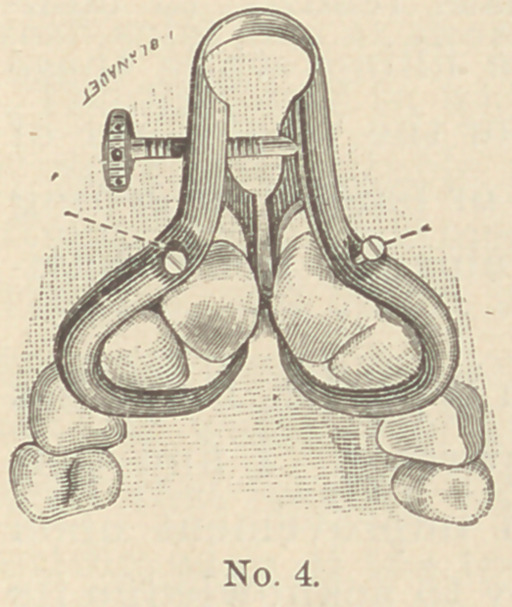


**No. 5. f5:**
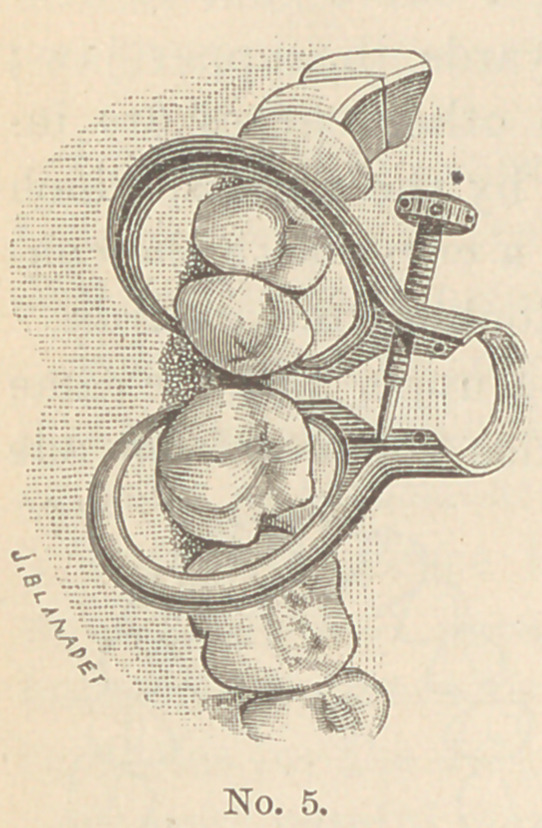


**No. 6. f6:**